# Impact of obesity on retrograde ureteroscopic approach

**Published:** 2012-06-18

**Authors:** M Drăguţescu, R Mulţescu, B Geavlete, B Mihai, E Ceban, P Geavlete

**Affiliations:** * “Sf. Ioan” Clinical Emergency Hospital, Department of Urology, Bucharest; ** “Nicolae Testemiţanu” USMF, Chişinău, Republic of Moldova

**Keywords:** retrograde ureteroscopy, prevalence of obesity, urolithiasis

## Abstract

**Introduction:** High-grade obesity raises some specific problems regarding the endourological approach. The aim of our study was to determine if this pathology might influence the outcome of retrograde ureteroscopy.

**Materials and methods:** We evaluated the outcome of 88 ureteroscopies performed in highly obese patients during the last 5 years. The data were compared with the results of 88 consecutive ureteroscopies performed in normal weight patients.

**Results:** The success rate in the study group was of 91% by comparison with 95% in the normal weight group. The use of flexible ureteroscopes was imposed in 17% of the obese group vs. 11% in the control group. The complications rate (all mild) was of 6.8% in the obese group vs. 4.5% in the normal weight patients. The differences between the two groups, although present, were not statistically significant. However, in two cases with obesity, the weight of the patients was too high for the operating table, imposing supplementary sustaining measures.

**Conclusions:** Ureteroscopic treatment of stones in obese patients is an acceptable treatment modality, with success rates similar to non-obese patients. Sometimes it may require some logistic measures in the operating theatre.

## Introduction

Obesity is a chronic disease with an increasing prevalence. According to the National Center for Health Statistics, 61% of the Americans are overweight (body mass index – BMI ≥ 25), 32% are obese (BMI ≥ 30) and 4.7% are morbidly obese (BMI ≥ 40) [**1**]. In the United States, the obesity rates increased by 48% in the last 15 years, while the incidence of morbid obesity grew from 2.9% (National Health and Nutrition Examination Survey 1988-1994) to 4.7% in 1999/2000, thus emphasizing a 62% increase in one decade.

This situation has major implications for co-morbid diseases, mortality rates and healthcare costs. Obesity is one of the leading preventable causes of death worldwide. In the United States, obesity is estimated to cause an excess of 111,909 to 365,000 deaths per year while in Europe 1 million deaths (7.7%) are attributed to the excessive weight. The healthcare costs related to obesity are of approximately $100 billion [**2**]. 

Even though data concerning obesity in Romania are controversial, statistics reveal that one third of the population suffers from obesity, which puts our country in Europe’s top ten classifications in this regard.

Obesity is generally considered a risk factor for surgery, as it is assumed that the complication rates are higher. Due to the physiological changes that alter cardiac, respiratory, metabolic and haemostatic functions, obesity predisposes to co-morbidity and increased risk [**3**]. Moreover, the increasing prevalence of obesity and morbid obesity suggests that the surgical treatment in these patients will become more common [**4**].

When treating urolithiasis, the use of lithotripsy, rigid and flexible ureteroscopy and percutaneous nephrolithotomy severely decreased the frequency of open surgery. These modern techniques are minimally invasive, safe and only require short in-patient hospital stays. High grade obesity pose special problems to the urologist, as the physical examination is more difficult, imaging is limited, special ‘heavy’ operating-tables may be required and patient positioning is often problematic. 

The present study aimed to determine if this pathology might actually influence the outcome of retrograde ureteroscopy (URS).

## Materials and methods

The last five years (2006-2011) charts were retrospectively reviewed in order to determine the efficacy and safety of the ureteroscopic treatment in a technically challenging group of patients with ureteral stones and a significant morbidity risk. The outcomes and complications of ureteroscopies performed in a group of highly obese patients were compared to those of a normal weight’ series of cases treated during the same period. 

The most common method of defining obesity is the Body Mass Index (BMI). BMI is a more objective measure of obesity than weight alone, because it accounts for height as well. The BMI also correlates with other measures of body fat and constitutes a readily available indicator of obesity. BMI measures the height to weight ratio by taking weight in kilograms and dividing it by height in squared meters (kg/m2).

According to the World Health Organization’ Guidelines, a BMI of 18.5 to 25 kg/m2 is considered normal, overweight is established by a BMI of 25 to 29.9, obesity is established at a BMI ≥ 30 and morbid obesity is present at a BMI ≥ 40 kg/m2.

The BMI for this series of patients was calculated by retrospectively examining the medical records. Any patient with a BMI ≥ 35 kg/m2 who was subjected to a retrograde ureteroscopy for ureteral stones was included in the study. Data regarding the preoperative characteristics, intraoperative features, outcomes and complications were collected. In total, 88 ureteroscopies performed in 83 obese patients were reviewed and compared with 88 consecutive ureteroscopies performed in 82 normal weight patients.

**Fig. 1:**
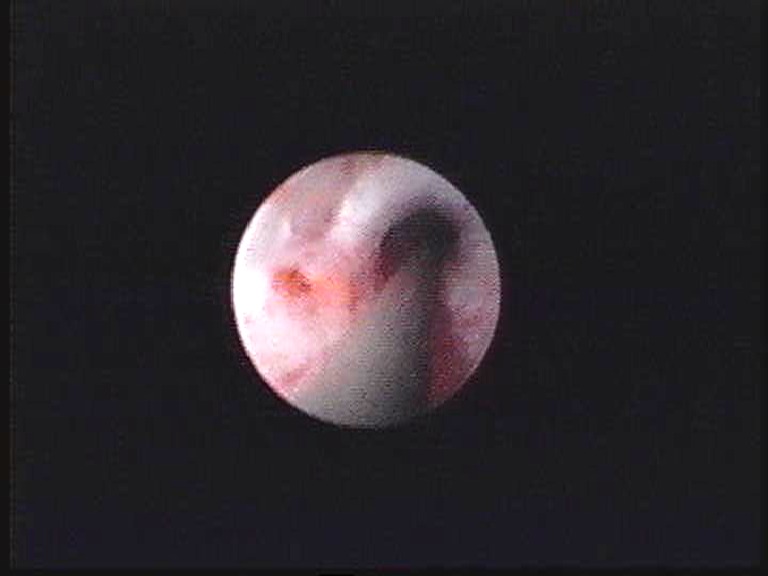
Semirigid ureteroscopic approach in an obese patient

**Fig. 2:**
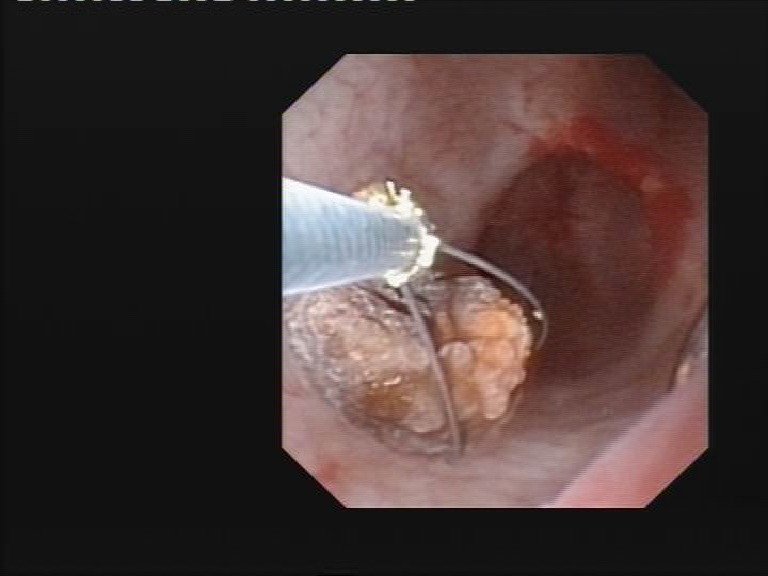
Flexible digital uretero-renoscopic approach in an obese patient

The Storz 10F semirigid and/or Olympus URF-Vo flexible ureteroscopes were used as required by each procedure (**Fig. 1,2**).

Our standard technique for the ureteroscopic treatment of ureteral calculi included cystoscopy with retrograde pyelogram, the placement of a 0.038-inch floppy-tipped guide wire past the stone (glide-wire when necessary) to maintain access, the mounting of a safety wire for flexible approach followed by the actual ureteroscopy with electro-hydraulic lithotripsy. Basket retrieval of stone fragments was employed when necessary. All procedures were performed under spinal anesthesia.

In order to document the stone size and location, KUB and abdominal ultrasound were performed in all patients, while intravenous pyelogram (IVP) and computer tomography (CT) were used when indicated. Patients were followed postoperatively by X-ray, CT and/or IVP until they became stone-free or received additional treatment.

The patients’ age, sex, stone size and outcome were recorded. The treatment outcomes were defined as radiographic evidence of fragmentation or complete stone disappearance. A procedure was considered successful when the postoperative imaging revealed fragments of 2 mm or less [**5**]. 

For each of the treatment groups, 95% confidence intervals were calculated for the treatment’s success rates. The statistical comparison of two independent percentages was done by means of the Fisher’s exact test (2-sided, p = 0.05). If the resulting p value was < 0.05, the difference in the sample percentages was considered statistically significant.

## Results 

A total of 88 retrograde ureteroscopies were performed in 83 obese patients (44 women and 39 men) with a BMI ≥ 35 kg/m2 in the study group. The mean age of the patients was 47 years (range 22 to 71 years old). The average weight was 122.3 kg (range 96 to 167 kg). The mean BMI was 38.8 kg/m2 (range 35.2 to 57.7 kg/m2). The overall mean individual stone size was of 8 mm, ranging from 4 to 20 mm.

A total of 88 retrograde ureteroscopies were performed in 86 normal weight patients (41 women and 45 men) in the control group. The average age was 49 years (range 20 to 79 years old). The mean BMI was 22.7 kg/m2 (range 19.8 to 24.9 kg/m2). Also, the overall mean individual stone size was of 9 mm, ranging between 5 and 22 mm. 

Data concerning the impact of obesity (BMI ≥35 kg/m2) on the stone-free rates were analyzed. Although their stone burdens were slightly smaller, yet not in a significant proportion (8 versus 9 mm, p=0.96), the stone-free rate for the ureter was of 91% in obese patients and 95.4% in normal weight cases (p = NS). No significant differences were found with regard to the success rates. 

Eight ureteroscopies in the study group and five in the control group were judged as failures (no stone-free status achieved). Practically, there were new renal calculi present postoperatively, consistent with the proximal migration of ureteral stone fragments that did not clear.

**Fig. 3:**
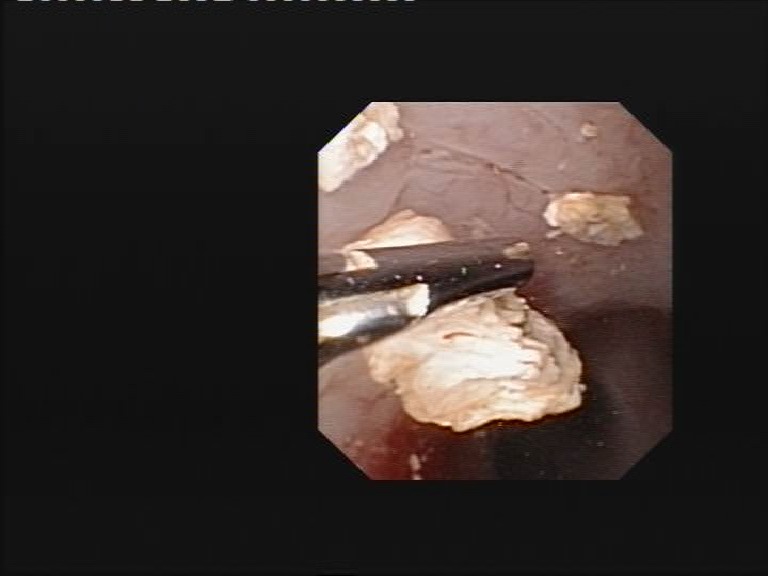
Stone fragments extraction through a flexible ureteroscope

The use of flexible ureteroscopy was imposed in 17% of the obese group versus 11% in the control study arm (**Fig. 3**).

The complication’ rate (all events of a mild intensity) was of 6.8% in the obese series versus 4.5% in normal weight patients. The differences between the two groups, although present, were not statistically significant. However, in two cases of morbid obesity, the patients’ weight exceeded the maximum weight supported by the operating table (150 kg), thus imposing supplementary sustaining measures.

## Discussion

From the very beginning, obesity causes diagnostic problems in the urological field. Symptoms of stone disease may be vague and pain is generalized, thus leading to diagnostic confusions. The physical examination is difficult. All imaging suffers from poor image quality: plain X-rays become scattered, renal ultrasound displays a beam’ attenuation while the kidney’s depth makes it difficult to find. CT machines have weight limits and special operating tables are often required.

The obese patient with urolithiasis presented two specific issues. The first one was constituted by the technical challenges of stone surgery related to the body habitus, while the second one was represented by co-morbidities affecting the anesthesia and actual operation in obese patients in general.

Few reports provided a comparison regarding the outcomes and complications of open stone surgery versus the endoscopic approach [**6**].

The intraoperative morbidity emphasized in obesity cases is related to anesthetic concerns, including altered pulmonary mechanics and oxygen perfusion. The obese patient may also have decreased preoperative cardiac parameters, susceptible to worsening function during surgery. Postoperatively, the greatest risks are those of thromboembolic complications and wound infection. 

Although PCNL in supine position is a viable alternative in such cases [**7**], there are some inconveniences which make the procedure possible in experienced centers alone (too short instruments, difficult positioning) [**8**]. However, without any doubt, the retrograde approach benefits from a reduced morbidity compared to PCNL. Due to these facts, ureteroscopy can overcome the shortcomings of PCNL and open surgery in obese patients [**9**].

The ideal treatment for ureteral stones is a rather controversial subject, with the non-invasive nature of extracorporeal shock wave lithotripsy (SWL) favored by many over URS. SWL was recommended as first-line treatment of ureteral calculi smaller than 1 cm, resulting in an up to 92.6% stone-free rate for proximal stones and 97.5% for mid and distal calculi. However, in obese patients, the use of SWL is often contraindicated due to the weight limits of SWL tables. Also, a frequent factor limiting the SWL’ success is the positioning of the patient in order to locate the stone at the focal point of the lithotripter [**10**], as the significantly increased skin-to-kidney depth impedes on the ability to focus the shockwaves. For this reason, SWL may not have the same success rates as in non-obese patients. Delakas et al. reported an increased chance of SWL failure in obese patients of 1.9 fold when the BMI was > 30 [**11**]. Also, Muñoz et al. found a 72% stone free rate secondary to SWL in these patients. Consequently, SWL may constitute a sub-optimal treatment for obese patients [**12**]. 

One of the greatest benefits of ureteroscopy is represented by its success rates unaffected by patient size, as shown in multiple studies [**13**], including our own. Satisfactory success rates are reported when compared to non-obese patients [**14,15**].

The improved success rates of URS may make it an essential treatment approach from the perspective of an increasingly obese population, with remarkably high stone-free rates.

## Conclusions

The ureteroscopic stone treatment in obese patients is an acceptable therapeutic modality, with success rates similar to those encountered in non-obese patients. In certain circumstances, specific logistic measures may be required in the operating theatre.
